# The Effect of Cadence on Shank Muscle Oxygen Consumption and Deoxygenation in Relation to Joint Specific Power and Cycling Kinematics

**DOI:** 10.1371/journal.pone.0169573

**Published:** 2017-01-06

**Authors:** Knut Skovereng, Gertjan Ettema, Mireille van Beekvelt

**Affiliations:** Centre for Elite Sports Research, Department of Neuroscience, Norwegian University of Science and Technology, Trondheim, NORWAY; Universita degli Studi di Roma 'Foro Italico', ITALY

## Abstract

The purpose of the present study was to investigate the effect of cadence on joint specific power and cycling kinematics in the ankle joint in addition to muscle oxygenation and muscle VO_2_ in the gastrocnemius and tibialis anterior. Thirteen cyclists cycled at a cadence of 60, 70, 80, 90, 100 and 110 rpm at a constant external work rate of 160.1 ± 21.3 W. Increasing cadence led to a decrease in ankle power in the dorsal flexion phase and to an increase in ankle joint angular velocity above 80 rpm. In addition, increasing cadence increased deoxygenation and desaturation for both the gastrocnemius and tibialis anterior muscles. Muscle VO_2_ increased following increased cadence but only in the tibialis anterior and only at cadences above 80 rpm, thus coinciding with the increase in ankle joint angular velocity. There was no effect of cadence in the gastrocnemius. This study demonstrates that high cadences lead to increased mVO_2_ in the TA muscles that cannot be explained by power in the dorsal flexion phase.

## Introduction

The external work rate produced during cycling is mainly generated by the muscles spanning the hip, knee and ankle joints [1]. Whereas the large mono articular muscles working over the hip and knee joints are regarded the main power producing muscles (i.e., the gluteus and the vasti), the role of the muscles working over the ankle joint (e.g., the tibialis anterior, gastrocnemius and soleus) are thought to transfer power to the crank and control the foot during the pedal stroke [2, 3]. The relative joint power contribution to the overall external work rate is affected by cadence, with increasing cadence leading to increased knee contribution and decreased hip contribution [4–6]. Contrary, the ankle joint’s contribution to external work rate is unaffected by changing cadence [4, 5, 7, 8].

The outcome at the joint is the result of the work done by multiple muscles, and therefore joint specific power may not provide a complete picture of the power contribution of the individual muscles. Additionally, the ankle movement during the pedal cycle consists of a plantar flexion phase and a dorsal flexion phase [5] and muscles associated with these two movements (e.g. tibialis anterior (TA) and gastrocnemius(GAS)) could potentially be affected differently by a change in cycling cadence. Furthermore, with regard to the previously mentioned ankle function, there can be other factors, such as range of motion (ROM) and joint angular velocity, that relate to the energy expenditure of the muscles spanning the ankle joint. An increased joint angular velocity at a joint would require the muscles spanning that joint to work at an increased contraction speed. Ankle ROM has been reported to decrease following increased cadence during maximal [9] and submaximal [10] pedalling, minimizing the effect on joint angular velocity [9]. In addition, a reduced ROM has been proposed as a strategy for simplifying the pedalling task by reducing joint angular velocity [9].

Changing cadence requires different movement speeds, and thereby likely affects muscle activation by changing the amount of recruited fast twitch fibres [11]. There are few studies that investigated the effect of cadence on muscle activation in the shank and there seems to be no consensus. Baum and Li [12] found an increased muscle activation with increasing cadence in the tibialis anterior (TA) and Neptune et al., found increasing GAS muscle activity with increasing cadence [13]. These differences could have resulted from the high interindividual variability that has been reported for muscle activity [14]. However, a difference in the amount of activity observed in a muscle does not necessarily mean that that muscle used more energy. Additionally, muscle activation does not distinguish between the various energy systems. Therefore, investigation of the metabolic requirement of individual muscles during exercise can help to elucidate the effect of cadence on the role of the individual muscles.

The few studies on the effect of cadence on muscle deoxygenation at a constant power output have reported both an effect [15] and no effect [16] in the vastus lateralis. However, to the best of our knowledge, no study has investigated the effect of cadence on oxygenation and local muscle oxygen consumption (mVO_2_) in the muscles of the shank during multi-joint whole-body exercise, such as cycling. Although the ankle joints relative contribution to external work production has been shown to be unaffected by changes in cadence [4, 5, 7, 8] the role of power transfer to the cranks [2] can potentially, and as indicated through EMG [12, 13], lead to a change in metabolic requirements of the shank muscles at changing pedalling frequency. Furthermore, the need for stabilization of the limbs at high cadence, as proposed by Boone et al. [17], may increase the metabolic requirements of the shank muscles.

Therefore, the purpose of this study was to investigate the effect of cadence on deoxygenation and mVO_2_ in the lateral gastrocnemius (GAS) and the tibialis anterior (TA) and to investigate the relationship between GAS and TA mVO_2_ and the joint specific power and cycling kinematics in the ankle. We hypothesised that increasing cadence would lead to a non-uniform effect on deoxygenation and mVO_2_ of the GAS and TA due to their involvement in plantar and dorsal flexion respectively. Additionally, we hypothesised that we would not find a relationship between joint specific power in the ankle and mVO_2_ in the TA and GAS muscles due to the proposed function of power transfer and stabilization as opposed to power production.

## Methods

### Subjects

Thirteen recreationally trained level 3 cyclists (age, 40±5 yr; body height, 184.2±5.16 cm; body mass, 81.7 ±7.4 kg; peak pulmonary oxygen consumption (VO_2peak_), 56.0 ± 4.9 ml/kg/min; peak power output, 372.1 ± 35.1 W and 4.6 ± 0.4 W/kg) participated in the study [18]. Permission to conduct the study was given by the Regional Ethics Committee for Medical and Health Research in Trondheim and a signed written informed consent was obtained from all participants prior to their participation.

### Experimental protocol

The participants visited the laboratory on three different days, each separated by one day of rest. The data collected for this study was part of a bigger study and therefore much of the methodological details have been previously published. Please see [6] for details. In brief, the first day consisted of the measurement of body mass, height, and skinfold thickness at the sites of the NIRS optodes. In addition, an extended occlusion test consisting of a 10-minute arterial occlusion was performed in a semi-supine position. The second day consisted of an incremental step cycling exercise test, starting at 100 W, with 25 W increments every 4 minutes until the blood lactate level exceeded 4 mmol/L. This was followed by an incremental cycling test with 25 W every minute for determination of peak power output and pVO_2peak_. The third day consisted of baseline oxygenation measurements of oxygenation followed by a controlled cadence test starting with a warm-up period of cycling at 50% of the work rate corresponding to a lactate concentration of 4 mmol/L (WR_4mmol_) at a freely chosen cadence followed by six 4-minute stages at increasing cadence (i.e. 60, 70, 80, 90, 100 and 110 rpm) at an external work rate corresponding to 75% of WR_4mmol_. Muscle oxygenation, heart rate and pulmonary oxygen consumption (pVO_2_) were measured continuously on all test days. Arterial occlusions were applied during the final 20 seconds of each stage of the controlled cadence test in order to measure mVO_2_. The participants continued pedalling during all arterial occlusions. Rating of perceived exertion and blood lactate were measured at the end of each stage of the controlled cadence test. Pedal force and kinematic variables were measured during 15-second periods, three times during each stage of the cadence controlled test. The subjects were unaware when pedal force and kinematic measurements were measured. The participants were instructed to cycle with a stable and constant trunk and hand position during all cycling in order to ensure consistent calculation of hip, knee and ankle angles.

### Instrumentation

Procedures and instrumentation were previously described in detail [6]. In brief, all cycling tests were performed on a cycle ergometer (Velotron, RacerMate inc, Washington, USA) in a laboratory with stable conditions. Measurements included blood lactate (Biosen C-Line Sport, EKF Industrial Electronics, Magdeburg, Germany), heart rate (Polar RS800, Polar Electro OY, Kempele, Finland), skinfold thickness (Holtain skinfold caliper, Holtain Ltd, Crymych, Wales) and pVO_2_ (Open-circuit indirect calorimetry, Oxycon Pro, Jaeger GmbH, Hoechberg, Germany, calibrated using gas from Riessner-Gase GmbH & Co, Lichtenfels, Germany and a 3-liter syringe). Muscle oxygenation was measured with a continuous wave near-infrared spectrophotometer system (Portamon, Artinis Medical Systems, the Netherlands) and an inflatable pneumatic cuff system (Hokanson E20 Rapid Cuff Inflator + Hokanson AG-101 Air Source, Marcom Medical ApS, Denmark) was used to apply the arterial occlusions.

Pedals instrumented with force cells (Revere Model 9363, capacity 250 kg per cell, the Netherlands) were used to measure pedal forces and a ProReflex 3D motion analysis system (Qualisys, Sweden) was used for kinematic analysis. A detailed description of the force pedal and kinematic analysis systems can be found in Ettema et al. [19] and Skovereng et al [6].

### Data analysis

Heart rate and pVO_2_ for each cadence stage was defined at the average of the last minute prior to the arterial occlusions. HR_peak_ was defined as the highest heart rate attained and pVO_2peak_ was calculated as the highest one-minute average attained during the maximum aerobic power-test.

Detailed descriptions of the calculations of joint powers for the lower limbs have been described previously [6], but in brief the calculations were done using inverse dynamics for a linked system of rigid segments [19–21]. Parameters for calculating masses and moments of inertia were taken from Van Soest et al. [22]. Range of motion (ROM) for the ankle joint was calculated as the difference between the minimum and the peak joint angle during the pedal cycle. Joint angular velocity was calculated as the mean absolute angular velocity averaged over the whole pedal cycle.

The methods for calculating deoxyhaemoglobin (HHb), oxyhaemoglobin (O_2_Hb), total haemoglobin (tHb), tissue saturation index (StO_2_) and mVO_2_ have been described in detail previously [6]. In brief, mVO_2_ was calculated from the linear change in HHb during arterial occlusion and the NIRS signal was corrected for changes in blood volume as described by Ryan et al. [23]. Changes in HHb, O_2_Hb and tHb are expressed as a change from rest in micromol, StO_2_ is expressed as a percentage of complete tissue oxygen saturation, and mVO_2_ is expressed relative to the physiological range obtained during ischemic calibration [23].

### Statistical analysis

Data are presented as mean ± standard deviation. Repeated measures ANOVA was used to evaluate the effect of cadence on blood lactate, whole body VO_2_, RPE, external work rate, ROM, joint angular velocity and ankle power. If the assumption of sphericity was violated, results were adjusted according to the Greenhouse-Geisser correction. A two-way ANOVA was used to evaluate the effect of cadence on joint specific power in the plantar flexion and dorsal flexion phase in addition to HHb, tHb, StO_2_ and mVO_2_ in the GAS and TA muscles. If the ANOVA was significant, *post hoc* analysis (Fishers LSD) was used to determine the effect of specific changes in cadence. Statistical significance was accepted at p < 0.05, and p < 0.1 was regarded as a tendency. Statistical analysis was performed using SPSS 22.0 (SPSS, Chicago, USA) for windows and Matlab R2013a (MathWorks inc. Natic, USA).

## Results

The self-chosen cadence during warm-up was 87.3 ± 9.0 rpm. The average external work rate measured at the pedals during all stages was 160.1 ± 21.3 W. External work rate did not differ between the various cadences. The increase in target cadence was met at all stages and no differences were found between target cadence and actual cadence (Table 1). The increase in cadence had a significant effect on pVO_2_, heart rate, blood lactate and rating of perceived exertion (all p < 0.05, Table 1).

**Table 1:** The effect of cadence on measured cadence, external work rate, pulmonary oxygen consumption, heart rate, blood lactate and RPE (rating of perceived exertion).

Targeted cadence	60	70	80	90	100	110
Cadence _(rpm)_	60.14±0.59	70.06±0.86*^,^^†^	80.25±0.79*^,^^†^	89.44±0.75*^,^^†^	98.67±1.67*	109.18±0.53*
External work rate _(w)_	158.7±21.2	159.5±20.6	161.4±20.1	160.1±22.4	160.1±21.1	160.6±22.6
pVO_2 (l/min)_	2.36±0.31 ^a^	2.40±0.29 ^a^	2.51±0.31 ^a,^*^,^^†^	2.62±0.28 ^a,^*^,^^†^	2.76±0.34 ^a,^*^,^^†^	2.95±0.36 ^a,^*^,^^†^
Heart rate _(%of peak)_	66.7±4.3 ^a^	68.7±4.9 ^a,^*^,^^†^	71.3.±4.7 ^a,^*^,^^†^	74.3±4.6 ^a,^*^,^^†^	77.7±5.1 ^a,^*^,^^†^	81.4±4.9 ^a^*^,^^†^
Blood lactate _(mmol/l)_	1.12±0.38	1.23±0.51*^,^^†^	1.42±0.53*^,^^†^	1.68±0.57*^,^^†^	2.18±0.81*^,^^†^	3.21±1.11*^,^^†^
RPE	11.7±0.68	12.2±0.79	12.4±0.70	12.9±1.1*	13.4±0.70*	14.1±0.88*

Values are mean ± standard deviation. pVO_2_ = pulmonary oxygen consumption, BLa = blood lactate, RPE = rating of perceived exertion.

* indicate a significant difference from a cadence of 60 rpm (p < 0.05).

^†^ indicate a significant change from previous cadence (p < 0.05).

**Fig 1:**
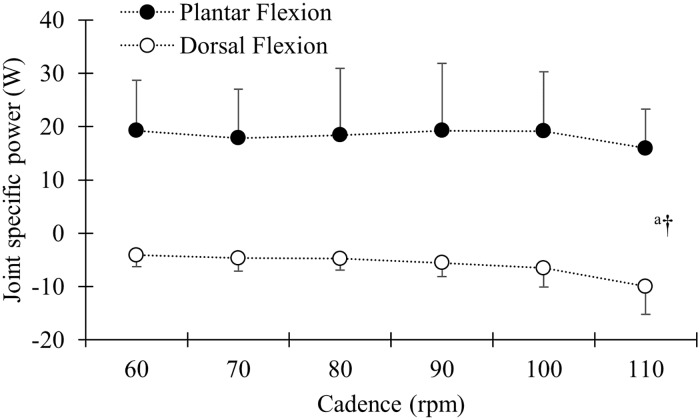
Group mean and standard deviation for plantar flexion (filled circles) and dorsal flexion (open circles) presented as absolute joint specific power in Watt (W). ^a^ indicate a significant main effect of cadence on joint power (p < 0.05). † indicate a main effect of phase on joint power.

No effect of cadence was found on ankle joint power averaged over the whole pedal cycle (p = 0.41). However, when plantar and dorsal flexion phases of the ankle movement were analysed separately (Fig 1), there was a main effect of cadence leading to decreased power (p < 0.05) and there was an effect of phase with more power produced in the plantar flexion phase (p < 0.05). There was no interaction effect of phase and cadence (p = 0.22).

**Fig 2:**
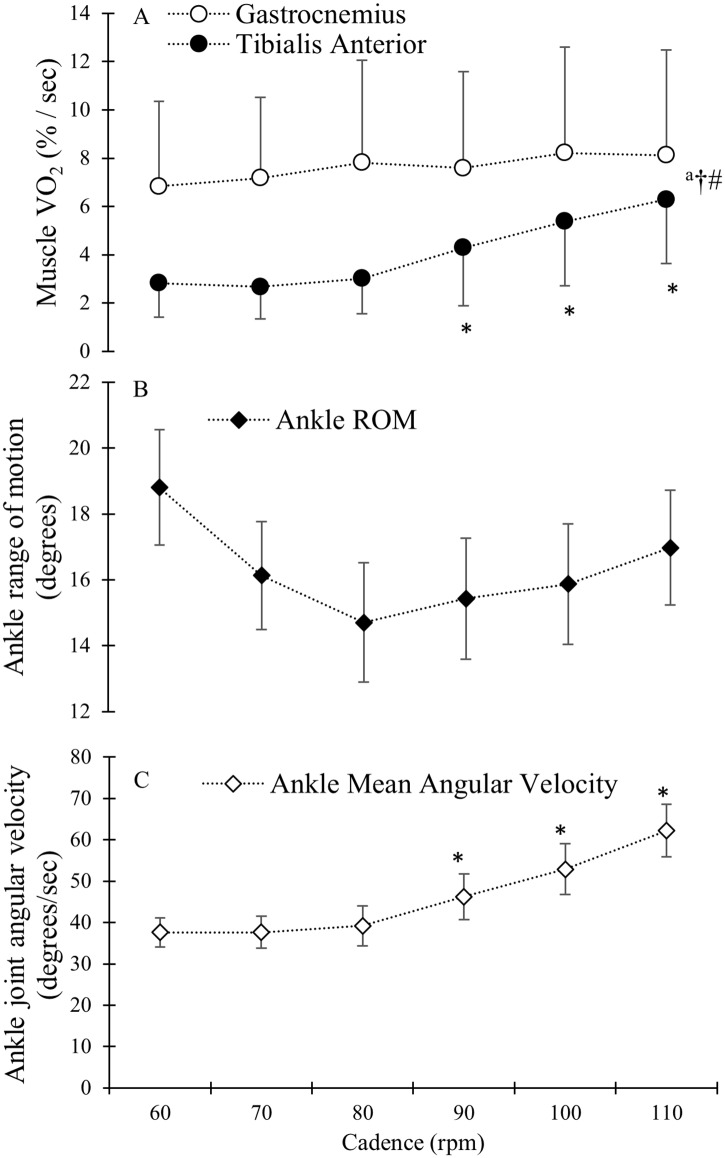
A) Group mean and standard deviation for muscle VO2 (mVO2) for the GAS (open circles) and TA (filled circles) presented as change in percent of ischemic calibration per second (% / sec). ^a^ indicates a significant main effect of cadence on mVO_2_ (p < 0.05). † indicate a main effect of muscle on mVO_2_. # indicate a significant interaction effect of cadence and muscle. * indicate a significant increase in mVO_2_ when compared to previous cadence. B) Ankle joint range of motion (filled diamonds) presented in degrees. C) Absolute ankle joint angular velocity (open diamonds) averaged over the whole pedal cycle in degrees / second.

For mVO_2_, there was a main effect of muscle (p < 0.05) and the overall mVO_2_ in the TA was lower than that in the GAS (Fig 2A). There was also a main effect of cadence (p < 0.05) with an increase in mVO_2_ at higher cadences. Additionally, a significant interaction effect was found, thus the effect of cadence on mVO_2_ was different for the TA and GAS muscles (Fig 2A, p < 0.05). TA mVO_2_ increased above 80 rpm whereas increasing cadence did not lead to a significant increase in GAS mVO_2_ (Fig 2A, p = 0.12).

Fig 2B shows changes in ankle ROM. There was a tendency for a decreasing ankle ROM (p = 0.06) with increasing cadence. Ankle joint angular velocity (Fig 2C) increased following increased cadence above 80 rpm (p < 0.05).

**Fig 3:**
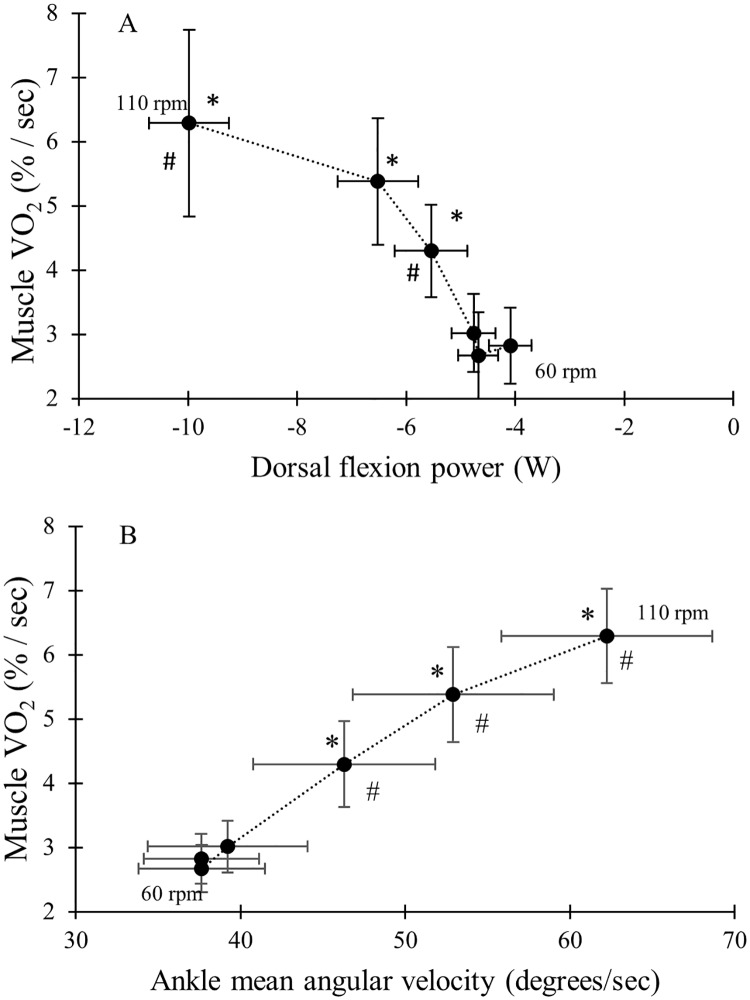
A) Mean and standard deviation for mVO_2_ (% / sec) of the TA presented as a function of ankle joint specific power in the dorsal flexion phase (W). * indicate significant differences in mVO_2_ from previous cadence. # indicate significant differences in power in the dorsal flexion phase from previous cadence. B) Mean and standard deviation for mVO_2_ (%/sec) of the TA presented as a function of ankle joint angular velocity (degree / sec). * indicate significant differences in mVO_2_ from previous cadence. # indicate a significant difference in ankle joint angular velocity from previous cadence.

The mVO_2_ in the TA, as a function of the ankle joint specific power in dorsal flexion phase, is shown in Fig 3A. Fig 3B shows TA mVO_2_ as a function of ankle joint angular velocity and demonstrates how the increase in mVO_2_ of the TA coincides with the increase in angular joint angular velocity.

**Fig 4:**
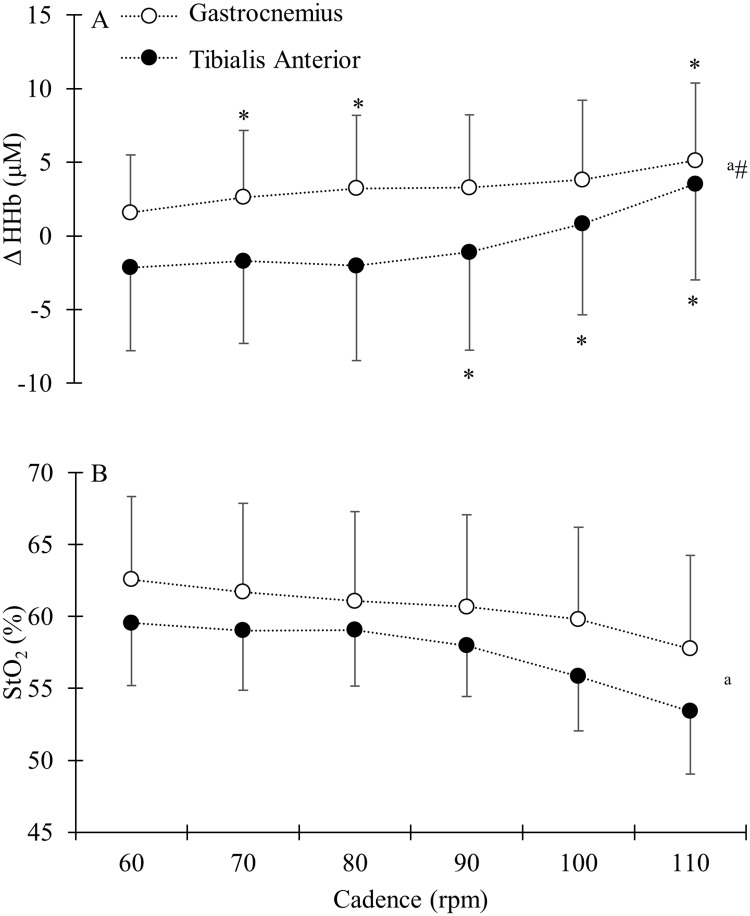
Group mean and standard deviation for A) muscle deoxygenation (HHb) presented as change from rest in micro molar and B) tissue oxygen saturation (StO_2_) presented as a percentage of complete tissue oxygen saturation for the GAS (open circles) and TA (filled circles). ^a^ indicate a significant main effect of cadence on HHb and StO_2_ (p < 0.05). # indicates a significant interaction effect of cadence and muscle. * indicate a significant change in HHb and StO_2_ from previous cadence (p < 0.05).

There was a tendency for a main effect of muscle for StO_2_ and HHb (Fig 4, p = 0.07 and 0.06 respectively). There was a main effect of cadence showing that increased cadence led to increased HHb and decreased StO_2_ in both the GAS and TA muscles (both p < 0.05). Additionally, a significant interaction effect between cadence and muscle was found for HHb (Fig 4, P < 0.05). Whereas HHb in the TA increased at cadences above 80 rpm, the effect on the GAS was much more gradual (Fig 4A). There was also a tendency for an interaction effect between the TA and GAS muscle for StO_2_ (p = 0.052).

For O_2_Hb there was no main effect of muscle (p = 0.34), but there was an effect of cadence (p < 0.05), similar to the effect on HHb, and leading to a decrease in O_2_Hb at the higher cadences (i.e. above 80 rpm). However, no interaction effect was found between muscle and cadence for O_2_Hb (p = 0.12). For tHb, there was no main effect of muscle (p = 0.19), no main effect of cadence (p = 0.13), and no interaction effect (p = 0.57).

## Discussion

To the best of our knowledge, this was the first study to investigate the effect of cadence on muscle oxygenation and mVO_2_ in the GAS and TA muscles during cycling at a constant external work rate. Additionally, the purpose of this study was to investigate the relationship between mVO_2_ and joint specific power in the ankle. The main finding was that increasing cadence led to increased mVO_2_ in the TA muscle which coincided with a decrease in ankle specific power in the dorsal flexion phase and an increase in ankle joint angular velocity. We found no change mVO_2_ in the GAS across cadences, which was not in accordance with changes in joint specific power.

We found no change in the ankle joint specific power, in accordance with the literature [4, 5, 7, 8]. However, when separated into plantar flexion and dorsal flexion, more power was produced in the plantar flexion phase compared to the dorsal flexion phase. Increasing cadence also led to a decrease in power in both phases which is in contrast to the findings of Ericson [8], likely due to the simultaneously increase in cadence and external work rate used in the study by Ericson [8]. Greater power in the plantar flexion phase compared to the dorsal flexion phase in addition to decreased power in the plantar flexion phase and increased negative power in the dorsal flexion phase when increasing cadence has been reported during maximal pedalling [9]. However, to the best of our knowledge, this was the first study that reported decreased plantar flexion power and increased negative power during dorsal flexion when cadence was increased during constant load moderate intensity pedalling.

The TA showed a significant increase in mVO_2_ at the higher cadences which also coincided with an increase in negative power during dorsal flexion, indicating power absorption, which are two findings in contradiction with each other [24]. Similarly, with regard to the GAS, the lack of a change in mVO_2_ was not in accordance with the decrease in power in plantar flexion from an energy perspective. However, increasing joint angular velocity could lead to an increase in the effort required of the stabilizing muscles as well as the muscles of the shank working at a higher contraction rate. Both aspects could result in increased mVO_2_. Indeed, an increased joint angular velocity in the ankle was found when cadence increased above 80 rpm, which coincided with increased TA mVO_2_. We found a tendency for reduction of ROM as cadence increased through the lower cadences, and although the reduction was not significant, it coincided with a constant joint angular velocity up to 80 rpm. By reducing ROM at increasing pedalling rate, the joint angular velocity might be kept constant and velocity of shortening might even be reduced [25]. The tendency of reduced ROM may be part of the stabilizing strategy which allows for greater force generation by muscle due to maintained shortening velocity and as a consequence, reduce energy expenditure. Previous studies have also reported reduced ROM at high compared to low cadences [9, 25] and reduced ROM following increased cadence has been proposed as a strategy for simplifying the pedalling task by stabilizing the joint and reducing the degrees of freedom [9]. Increased deoxygenation in the vastus lateralis following increased cadence has been reported previously [17] and was also proposed to arise from an increased need for stabilization and altered contraction rates [17, 26]. The need for stabilization may possibly place higher demands on the stabilizing muscles at higher joint angular velocities as inertial forces from the shank increase at higher cadence which would subsequently require increased co-contraction of the shank muscles, increasing energy expenditure. However, while there could be an increased cost of stabilization, the decreased power during the plantar flexion phase offsets this, resulting in a zero net effect as is reflected by the stable GAS mVO_2_. Another possible explanation for the observed differences between the effect of cadence on GAS and TA mVO_2_ might be that the GAS has a primary role in power transfer and the TA has a primary role in stabilization.

An interesting observation was that the cadence where joint angular velocity and TA mVO_2_ initially increased (i.e. above 80 rpm) coincided with the first cadence above the freely chosen cadence measured at warm up (i.e. ~87 rpm). For highly skilled cyclists, high cadences have been shown to be more efficient [27] i.e. they do not show an increase in pVO_2_ with increasing cadence. It is possible that the use of high cadences in training by professional riders has made them better at controlling the pedalling action at high cadences. This indicates that the freely chosen cadence may be limited by the highest cadence where the cyclists are able to effectively control their foot during pedalling. However, whether this reduces the energy expenditure from lower limb or non-lower limb sources remains to be investigated.

In accordance with previous reports [18], we found that increasing cadence while cycling at a constant external work rate increased pVO_2_, heart rate and rating of perceived exertion. The question remains what causes the increase in pVO_2_. Umberger et al. [28] showed, using computer simulation, that during constant load cycling, lower limb muscles only increased their energy expenditure substantially at high cadences (i.e. at 120 rpm). Our results indicate that this also applies to oxygenation, saturation and mVO_2_ at high cadence, with the non-uniform effect of cadence visible only at the highest cadences. The increase in TA mVO_2_ contributes to the increase in pVO_2_ that occurs following increasing cadence in addition to the energy expenditure increase associated with non-lower limb muscle action [28].

The increase in HHb and decrease in StO_2_ with increasing cadence would be expected to increase mVO_2_. However, this was only found for the TA and not for the GAS. A potential explanation for the lack of an increased mVO_2_ in the GAS may be that HHb and StO_2_ depend on mVO_2_ and oxygen delivery. If both oxygen delivery and mVO_2_ in a muscle decrease, the saturation of that muscle could, in theory, remain unchanged. A decrease in oxygen delivery at the same mVO_2_, would lead to a decreasing saturation and oxygenation. We found a decrease in oxygenation with no change in mVO_2_ for the GAS and conclude, therefore, that oxygen delivery might have been impaired at the highest cadences. We found no effect of cadence on blood volume changes in the GAS. Blood volume changes, as measured by NIRS, do not directly reflect changes in muscle blood flow. Therefore, based on the current data, we cannot conclude whether or not blood flow was impaired at the highest cadences. The results of this study, looking at energy expenditure, showed clear differences between muscles of the shank, but it remains to be seen if the effect of cadence on oxygenation, mVO_2_ and blood volume is also influenced by training status and cycling experience as reported for muscle activity [29].

### Methodological considerations

The arterial occlusion method was used to calculate mVO_2_, a method which relies on constant blood volume within the measured tissue during the occlusions. A manual check of the NIRS signals revealed that this was not always the case. The signals were, therefore, corrected for changes in blood volume, in accordance to Ryan et al. [23], prior to mVO_2_ calculation. In addition, mVO_2_ was expressed as a change in individual ischemic calibration, as this has been reported to minimize the confounding effect of adipose tissue thickness [30].

In order for the measurements of mVO_2_ to be comparable to joint power, we needed to minimize the contribution of anaerobic metabolism during the test. Therefore, moderate intensity exercise was used in this study, and measurements with corresponding RER values above 1.0 and blood lactate values above 4.0 were not included. Heterogeneity of mVO_2_ within the vastus lateralis muscle has been reported previously [31] and since we only used one optode on each of the two muscles in the present study, there is a possibility that such heterogeneity has influenced our results.

No randomization of cadences was performed in the present study. This approach was taken due to the known increase in pVO_2_ [18] of increasing cadence and thus the hardest effort would come last in the test and minimizing its effect on subsequent cadences. Since the participants were cyclists and accustomed to training for extended periods of time, it was our belief that any fatiguing effects due to the duration of the protocol were minimal.

In conclusion, the present study demonstrates a significant effect of cadence on oxygenation and mVO_2_ that differs between the TA and GAS muscles. The effect of cadence on mVO_2_ in the TA coincides with an increased negative dorsal flexion power and an increase in the joint angular velocity of the ankle at cadences above freely chosen. These findings indicate that high cadences lead to increased mVO_2_ in the TA muscles which cannot be explained by an increase in power in the dorsal flexion phase and thus must be due to other mechanisms, such as greater energy requirements for controlling foot motion at higher angular velocity.

## Supporting Information

S1 File Previous publication from the same experiment. (PDF)

S1 Dataset Dataset for the present publication. (XLSX)
