# Supplementary material for: The Effect of Cadence on Shank Muscle Oxygen Consumption and Deoxygenation in Relation to Joint Specific Power and Cycling Kinematics
Source: PLoS One. 2017 Jan 6;12(1):e0169573. doi: 10.1371/journal.pone.0169573 (PMC5217861; doi:10.1371/journal.pone.0169573)
Supplement: S1 File — (PDF) [file pone.0169573.s001.pdf]

Eur J Appl Physiol
DOI 10.1007/s00421-016-3379-x

ORIGINAL ARTICLE

Oxygenation, local muscle oxygen consumption and joint specific power in cycling: the effect of cadence at a constant external work rate

Knut Skovereng[1] · Gertjan Ettema[1] · Mireille C. P. van Beekvelt[1]

Received: 12 October 2015 / Accepted: 11 April 2016
© The Author(s) 2016. This article is published with open access at Springerlink.com

Abstract
Purpose The present study investigates the effect of cadence on joint specific power and oxygenation and local muscle oxygen consumption in the vastus lateralis and vastus medialis in addition to the relationship between joint specific power and local muscle oxygen consumption (mVO_2).
Methods Seventeen recreationally active cyclists performed 6 stages of constant load cycling using cadences of 60, 70, 80, 90, 100 and 110 rpm. Joint specific power was calculated using inverse dynamics and mVO_2 and oxygenation were measured using near-infrared spectroscopy.
Results Increasing cadence led to increased knee joint power and decreased hip joint power while the ankle joint was unaffected. Increasing cadence also led to an increased deoxygenation in both the vastus lateralis and vastus medialis. Vastus lateralis mVO_2 increased when cadence was increased. No effect of cadence was found for vastus medialis mVO_2.
Conclusion This study demonstrates a different effect of cadence on the mVO_2 of the vastus lateralis and vastus medialis. The combined mVO_2 of the vastus lateralis and medialis showed a linear increase with increasing knee joint specific power, demonstrating that the muscles combined related to power generated over the joint.

Communicated by Carsten Lundby.

✉ Knut Skovereng
knut.skovereng@ntnu.no

[1] Department of Neuroscience, Centre for Elite Sports Research, NTNU, Norwegian University of Science and Technology, Trondheim 7491, Norway

Keywords Near-infrared spectroscopy · Muscle VO_2 · Joint specific power · Cycling · Cadence

Abbreviations

AO	Arterial occlusion
HHb	Deoxyhaemoglobin
MAP	Maximal aerobic power
mVO_2	Muscle oxygen consumption
NIRS	Near-infrared spectroscopy
O_2Hb	Oxyhaemoglobin
pVO_2	Whole body oxygen consumption
StO_2	Tissue saturation index
tHb	Total haemoglobin
VL	Vastus lateralis
VM	Vastus medialis
WR_{lt}	Work rate at lactate threshold

Introduction

Cycling research has focused much on the effect of cadence on cycling energetics (Ettema and Loras 2009). With the exception of low cadence (<60 rpm), there is a trend for the metabolic cost to increase with increasing cadence, and there seems to be an optimal cadence which increases with increasing work rate (Foss and Hallen 2004). Despite this, the most energetically efficient cadence at a specific external work rate has been shown to be lower than the self-chosen cadence by cyclists (Foss and Hallen 2004; Lucia et al. 2004). However, other factors than whole body metabolic cost plays a role in how we pedal.

Apart from a change in metabolic cost, changing cadence also leads to technical responses, such as alternations in muscle activation and force effectiveness, among others (Ansley and Cangley 2009; Ettema and Loras 2009;

Hug and Dorel 2009; Leirdal and Ettema 2011). Whereas the large mono articular muscles working over the hip and knee joints are regarded as the main power producing muscles (i.e., the gluteus and the vasti), the role of the muscles working over the ankle joint is also to transfer this power to the crank (Zajac 2002). The effect of both cadence and work rate on the relative joint contribution to external power, from the hip, knee and ankle, has been studied as well (Bini et al. 2010a; Elmer et al. 2011; Ericson 1988; Mornieux et al. 2007). The previous investigations regarding the effect of cadence on the relative joint power contribution to external work are not all in agreement, with some studies reporting no effect of cadence (Bini et al. 2010a; Ericson 1988) and some studies reporting increasing knee joint and decreasing hip joint contribution with increasing cadence (Elmer et al. 2011; Mornieux et al. 2007). All studies do agree that relative ankle contribution to external power is largely unaffected across cadences (Bini et al. 2010a; Elmer et al. 2011; Ericson 1988; Mornieux et al. 2007) with the exception of one study reporting increased ankle contribution (Bini et al. 2010b).

Going one step further, the joint power is a result from the action of multiple muscles. Most research on the effect of cadence on individual muscles has been focusing on the electrical activity of the muscle, as measured by EMG. However, the effect of cadence on the EMG results are ambiguous. Muscle activation of the vastus lateralis (VL) has been shown to decrease with increasing cadence in elite level cyclists (Lucia et al. 2004), while it remained stable in recreational cyclist (Sarre et al. 2003). In contrast, Neptune et al. (1997) found increased muscle activation in the vastus medialis (VM) following increased cadence. While EMG can provide indirect information about the magnitude of muscle force, it does not distinguish between the various energy systems required to initiate muscle contraction and force production. Additionally, an increase in cadence also results in increased movement speed, thereby affecting muscle recruitment by an increased involvement of fast twitch fibres (Sargeant 2007). To obtain an indication of the aerobic energy consumption, near-infrared spectroscopy (NIRS) can be used.

Near-infrared spectroscopy (NIRS) is a non-invasive, continuous method that can be used to measure concentration changes in oxygenated and deoxygenated haemoglobin and myoglobin (similarities in absorption spectra result in an inability to differentiate between haemoglobin and myoglobin). This allows us to investigate the balance between O_2 delivery and extraction at the muscle level (Boone et al. 2014; Murias et al. 2013) and changes in deoxyhaemoglobin (HHb) and tissue saturation index (StO_2) are thought to represent changes in the oxygen extraction of the microvasculature (DeLorey et al. 2003). Total haemoglobin (tHb) is calculated by summing HHb and oxyhaemoglobin (O_2Hb) and is used to evaluate changes in blood volume within the active tissue. Additionally, by applying an arterial occlusion (AO), the rate of change in HHb allows us to calculate local muscle oxygen consumption (mVO_2).

Few studies have investigated the effect of cadence on VL muscle oxygenation during incremental external work rates and no clear agreement has been found (Boone et al. 2014; Ferreira et al. 2006). Also, investigations on the effect of cadence on muscle oxygenation at a constant power output show no clear agreement, reporting both an effect and no effect of cadence (Takaishi et al. 2002; Zorgati et al. 2013). Additionally, Zorgati et al. (2013) reported only HHb, Takaishi et al. (2002) reported HHb and tHb, but none of these studies investigating oxygenation during constant load exercise reported StO_2. To the best of our knowledge, only one study has studied the effect of cadence on StO_2 during constant load cycling exercise (Kounalakis and Geladas 2012) and reported no effect. However, the study by Kounalakis and Geladas (2012) only used two cadences and only focused on the VL.

Although there is no complete consensus with regard to muscles oxygenation and joint specific power, if we investigate the oxygenation and mVO_2 response of a local muscle in relation to a joint specific measure of work rate, we would expect a linear relationship. Two issues are important when investigating this relationship. The mVO_2 of biarticular muscles would be hard to interpret because they work over two joints. Additionally, the outcome at the joint level is made up of multiple muscles. Therefore, measuring various mono-articular muscles working over the same joint would give a more accurate estimate of muscle energy consumption at one joint.

Examination of the joint specific power contribution and the corresponding metabolism in specific muscles coupled to each joint would help understand possible changes in the coordinative patterns and local muscle energetics in cycling at different cadences (Bini and Diefenthaeler 2009; Ericson 1988). The purpose of the present study was therefore, to investigate the effect of cadence on the joint specific contribution to external power and the relationship between joint power and deoxygenation and mVO_2 in the VL and VM during constant load cycling exercise.

Methods

Subject

Seventeen recreationally trained cyclists (mean ± standard error: age 40.6 ± 1.4 years; height 181.4 ± 1.2 cm; body mass 81.8 ± 1.2 kg; pVO_{2peak}, 55.1 ± 1.2 ml/kg/min;

maximum aerobic power, 358.8 ± 7.4 W), thus categorized as level 3 cyclists (Ansley and Cangley 2009), participated in the study. We were given permission to conduct the study by the regional ethical committee and signed written informed consent was obtained from all participants prior to their participation in the study.

Experimental protocol

The participants came to the laboratory on three separate days. On the first day an arterial occlusion test consisting of a 10-min arterial occlusion (AO) was performed. The AO was performed during rest in a semi-supine position with an inflatable cuff placed on the upper right thigh. The second day started with resting measurements of mVO_2, blood lactate, heart rate and pVO_2. Resting measurements were followed by an incremental test, which started with 4 min of unloaded cycling followed by an incremental exercise protocol, starting at an external work rate 100 W, with 25 W increments every 4 min until the blood lactate level exceeded 4 mMol/l. Blood lactate was measured at the end of each work rate and a freely chosen cadence was used. The external work rate corresponding to 4 mMol/l was used as lactate threshold (WR_{lt}). After a period of ~5 min using active recovery, a maximal aerobic power test (MAP-test) was conducted. The MAP-test consisted of an incremental test using 25 W/min increments in external work rate until voluntary exhaustion at a freely chosen cadence. The third day consisted of a controlled cadence test starting with a warm up period of cycling at 50 % of WR_{lt} at a freely chosen cadence followed by six 4-min stages at different cadences (i.e., 60, 70, 80, 90, 100 and 110 rpm) at a work rate corresponding to 75 % of WR_{lt}. The low external work rate was chosen in order to ensure predominantly aerobic metabolism. Muscle oxygenation, heart rate and pVO_2 were measured continuously during all tests. Rating of perceived exertion and blood lactate were measured at the end of each stage of the controlled cadence test. Pedal force and kinematic variables were measured during 15-second periods, three times during each stage of the cadence controlled test. The subjects were not aware of the exact periods for pedal force and kinematic measurements. In order to measure muscle oxygen consumption, an AO was applied during the final 20 s of each stage of the cadence controlled test. The participants continued pedalling during the AO.

Skinfold thickness was measured at the first day of testing at the sites of the NIRS optodes. Body mass and height were measured on the first day of testing. The participants were instructed to cycle with a stable and constant trunk and hand position during all cycling tests in order to enable accurate calculation of changes in hip, knee and ankle angles.

Measurements and equipment

All measurements were conducted in a laboratory with steady conditions (temperature ~22 °C and relative humidity ~45 %). All cycling tests were performed on a cycle ergometer with constant external work rate (Velotron, RacerMate Inc., Washington, USA). Blood lactate was measured in 20 µl blood samples using the Biosen C-Line Sport lactate measurement system (EKF Industrial Electronics, Magdeburg, Germany). Heart rate was measured with a heart rate monitor (Polar RS800, Polar Electro OY, Kempele, Finland). Skinfold thickness was measured using a skinfold calliper (Holtain skinfold calliper, Holtain Ltd, Crymych, Wales).

An open-circuit indirect calorimetry apparatus was used to measure pVO_2 (Oxycon Pro, Jaeger GmbH, Hoechberg, Germany). The equipment was calibrated on each day of testing using a 3-liter syringe and a gas of known concentration (16.0 % O_2 and 5.85 % CO_2, Riessner-Gase GmbH & Co, Lichtenfels, Germany).

Muscle oxygenation was assessed using two continuous wave near-infrared spectrophotometer systems (Oxymon MKIII and Portamon, Artinis Medical Systems, the Netherlands). The two systems were used simultaneously in order to measure two muscles in the right thigh [i.e., vastus lateralis (VL) and the vastus medialis (VM)]. Both systems had 3 transmitters and one receiver. For congruence in measurement depth, the inter-optode distance of 35 mm was used for further analysis of the concentration changes in oxy- and deoxyhaemoglobin/myoglobin. All transmitters emitted two wavelengths, which were 766 and 856 nm for the oxymon system and 762 and 841 nm for the portamon system. After the optode sites had been shaved, the optodes were placed on the muscle belly of the VL and VM muscles and secured with double-sided tape and elastic bandages. To ensure similar day-to-day optode placement, the sites of optode placement were marked on the first day of testing. An inflatable pneumatic cuff system (Hokanson E20 Rapid Cuff Inflator + Hokanson AG-101 Air Source, Marcom Medical ApS, Denmark) was used to apply the AOs. The cuff, placed on the top of the right thigh, was rapidly inflated (i.e. <0.5 s) to a pressure of 300 mmHg and rapidly deflated after 20 s of AO. An electrical signal corresponding to cuff inflation was used to synchronize the two NIRS systems.

Pedal forces were measured with pedals equipped with two force cells (Revere Model 9363, capacity 250 kg per cell, the Netherlands) capable of measuring vertical and horizontal forces with a sample rate of 500 Hz. The pedals were calibrated by applying shear and normal forces using weights of different magnitudes fastened on the pedals in directly vertical and horizontal directions. A detailed description of the force pedal system can be found in

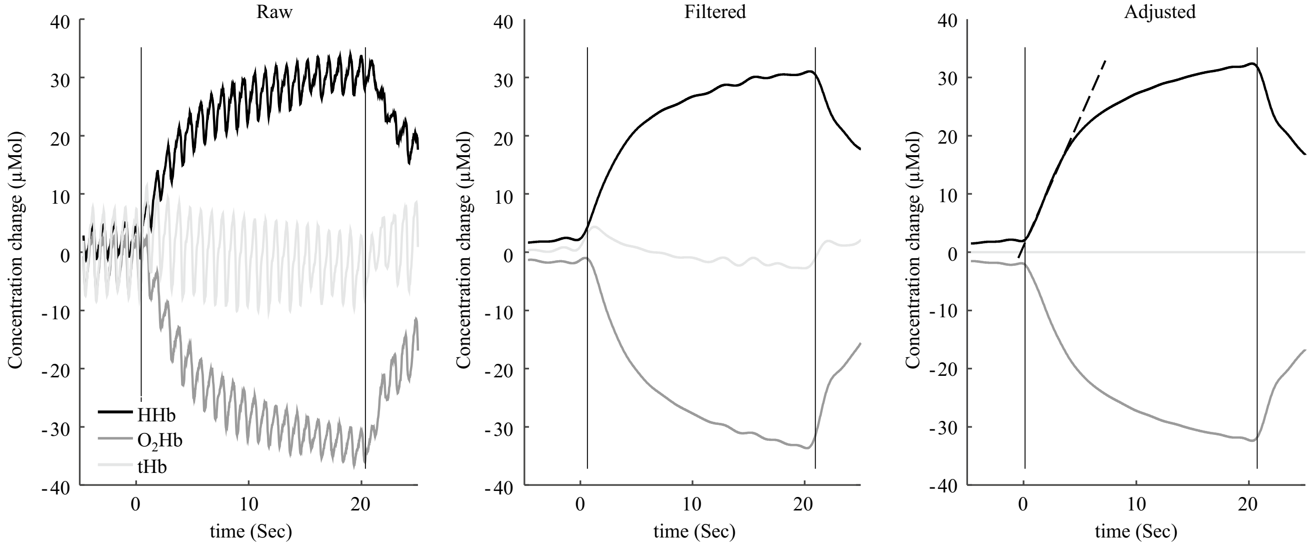

Fig. 1 Examples of the raw, filtered and the corrected NIRS signal for oxyhaemoglobin (*dark gray*), deoxyhaemoglobin (*black*) and total haemoglobin (*light gray*) in the VL during one AO cycling at a cadence of 60 rpm. *Vertical lines* indicate start and stop of the AOs and the *dashed line* show linear regression analysis used for calculation of m$\dot{V}O_2$ during the initial phase of occlusion

Ettema et al. (2009). Cycling kinematics were measured using a ProReflex 3D motion analysis system (Qualisys, Sweden) with eight cameras. Reflective markers were placed on the hip (greater trochanter), knee (lateral epicondyle) and ankle (lateral malleolus) joints and on custom made extensions placed symmetrically on the pedal axis.

Data analysis

Heart rate for each work load was defined at the average of the last minute prior to the AOs. HR_{peak} was defined as the highest heart rate attained during the MAP-test. p$\dot{V}O_2$ was averaged over the last minute of each work load of the lactate threshold test and p$\dot{V}O_{2peak}$ was defined as the highest one-minute average attained during the MAP-test. Joint powers for the legs (i.e., hip, knee and ankle joints) were calculated using inverse dynamics for a linked system of rigid segments (Ettema et al. 2009; Hull and Jorge 1985; McGhie and Ettema 2011). Parameters for calculating masses and moments of inertia were taken from Van Soest et al. (1993). Joint powers were averaged over all complete crank cycles during each of the 15 s recordings of kinematic and kinetic measurements and are expressed as the absolute power for each joint. Additionally, knee joint power was calculated specifically for the flexion and extension phases of joint action (Martin and Brown 2009). Joint power results are presented for the right leg.

Tissue saturation (StO_2), deoxyhaemoglobin (HHb), oxyhaemoglobin (O_2Hb) and total haemoglobin (tHb) for each cadence were calculated as the average value during the final 30 s prior to each of the AOs. HHb, O_2Hb and tHb are expressed as change from resting values in micromolar. StO_2 is expressed in percentage of saturation.

Local muscle oxygen consumption (m$\dot{V}O_2$) was calculated as the slope of linear change in HHb during the AOs using simple linear regression. The calculation of m$\dot{V}O_2$ is based on the assumption that blood volume is constant during the AOs. Due to forceful contractions during cycling it is difficult to be certain that the assumption holds, thus, the NIRS signal was corrected for changes in blood volume according to Ryan et al. (2012) prior to m$\dot{V}O_2$ calculation. Example plots of raw, filtered and corrected NIRS signal is shown in Fig. 1. Since we were using two NIRS systems to measure m$\dot{V}O_2$ is expressed as rate of change relative to the physiological range from ischemic calibration (Ryan et al. 2012). We report absolute values of m$\dot{V}O_2$ in ml/100 g/min calculated according to van Beekvelt et al. (2001a) for comparison purposes. We cannot distinguish between aerobic and anaerobic metabolism contributions to joint specific power. Therefore, in order to minimize the anaerobic metabolism during the m$\dot{V}O_2$ calculations, only measurements with corresponding blood lactate <4.0 mMol/l and RER values <1.0 were included for m$\dot{V}O_2$ calculation. Additionally, all regression analyses were quality checked and because m$\dot{V}O_2$ is based on an assumption of linearity during calculation, only regressions with a R^2 of 0.98 or better were included for further analysis.

Statistical analysis

The descriptive data are presented as mean ± standard error. The effect of cadence on absolute joint power,

Table 1 Group mean and standard error for external work rate, Cadence, pVO_2, Heart rate, blood lactate and rating of perceived exertion (RPE)

Target cadence (rpm)	60	70	80	90	100	110
External work rate $_{(W)}$	154.1 ± 4.67	154.2 ± 4.51	155.6 ± 4.74	155.3 ± 4.73	157.2 ± 4.48	156.6 ± 5.14
Cadence $_{\text{measured (rpm)}}$	60.4 ± 0.21	$70.1 \pm 0.28^{\#,*}$	$80.0 \pm 0.15^{\#,*}$	$89.6 \pm 0.25^{\#,*}$	$98.7 \pm 0.39^{\#,*}$	$109.4 \pm 0.13^{\#,*}$
pVO_2 $_{\text{(l/min)}}$	2.31 ± 0.07	2.37 ± 0.06	$2.49 \pm 0.06^{\#,*}$	$2.60 \pm 0.06^{\#,*}$	$2.74 \pm 0.06^{\#,*}$	$2.95 \pm 0.07^{\#,*}$
Heart rate $_{\text{(\% of peak)}}$	64.6 ± 0.85	$66.6 \pm 0.80^{\#,*}$	$68.7 \pm 0.88^{\#,*}$	$71.4 \pm 0.97^{\#,*}$	$74.7 \pm 1.09^{\#,*}$	$78.2 \pm 1.04^{\#,*}$
Blood lactate $_{\text{(mMol/l)}}$	1.06 ± 0.06	1.14 ± 0.08	$1.29 \pm 0.08*$	$1.54 \pm 0.11^{\#,*}$	$1.99 \pm 0.15^{\#,*}$	$2.88 \pm 0.24^{\#,*}$
RPE	11.3 ± 0.30	12.0 ± 0.24	12.2 ± 0.24	$12.5 \pm 0.26^{\#}$	$13.1 \pm 0.32^{\#}$	$13.5 \pm 0.43^{\#}$

# Indicates a significant change from a cadence of 60 rpm ($p < 0.05$)

* Indicates a significant change from previous cadence ($p < 0.05$)

relative joint power contribution and pVO_2 were evaluated using repeated measures ANOVA. If the assumption of sphericity was violated, results were adjusted according to the Greenhouse-Geisser correction. When significant main effects of work rate were found, post hoc analysis was performed using Bonferroni corrections to evaluate differences between cadences. Difference between resting condition and/or post warm up for HHb, StO_2, O_2Hb, tHb and mVO_2 was evaluated using pair-samples *t* test.

We evaluated differences in the effect of cadence on HHb, O_2Hb, StO_2 and mVO_2 between the VL and VM with two-way repeated measures ANOVA. The −2 Log likelihood ratio test was used to evaluate if the relationship between mVO_2 and joint specific power was best described by a linear or quadratic model. Statistical significance was accepted at $p < 0.05$ and all statistical analyses were performed using SPSS 22.0 (SPSS, Chicago, USA) for windows and Matlab R2013a (MathWorks inc. Natic, USA).

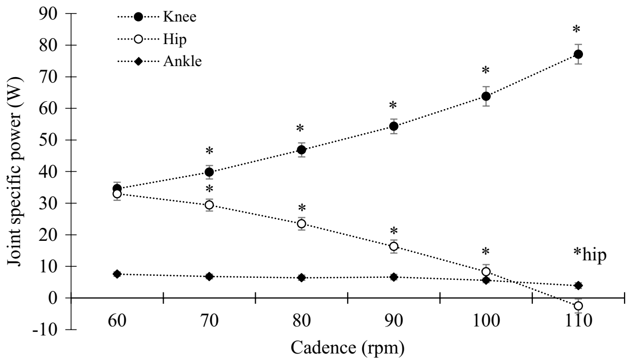

Fig. 2 Group mean and standard error for hip (*filled circles*), knee (*open circles*) and ankle joint (*filled diamonds*) presented as absolute joint specific power. *Asterisk* indicate a significant change in hip and knee joint specific power from previous cadence ($p < 0.05$). No change was found in ankle joint specific power

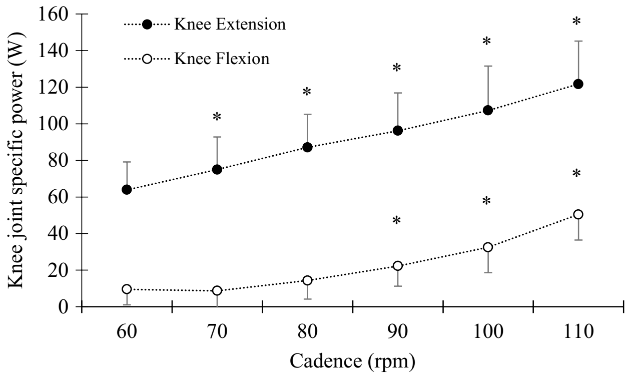

Fig. 3 Group mean and standard error for knee extension (*filled circles*) and knee flexion (*open circles*) presented as absolute joint specific power. *Asterisk* indicates a significant change in joint power from previous cadence ($p < 0.05$)

Results

The freely chosen cadence during warm-up was 88.6 ± 2.3 rpm. The external work rate calculated from pedal forces did not differ from the target work rate (i.e., 75 % of WR_{lt}) set on the ergometer (168.2 ± 4.7 W). The external work rate used during the cadence test corresponded to 47.0 ± 0.9 % of MAP and led to a pVO_2 ranging from 51.7 ± 1.2 to 66.2 ± 1.2 % of VO_{2peak} at 60 and 110 rpm, respectively. The actual cadence was not significantly different from the target cadence during any of the conditions (Table 1). The effect of cadence on pVO_2, blood lactate, heart rate and rating of perceived exertion, in addition to the actual cadence and external work rate calculated from pedal forces is presented in Table 1. For both pVO_2 and blood lactate, there was no change following the first cadence increase, but subsequent increases from 70 to 110 rpm led to a significant increase in pVO_2 (all $p > 0.05$). The rating of perceived exertion (RPE) was significantly increased at 90, 100 and 110 rpm compared to 60 rpm ($p > 0.05$). Heart rate increased following all increases in cadence (all $p > 0.05$). There was no difference between VL and VM skinfold thickness (10.2 ± 1.0 and 11.3 ± 0.9 mm respectively, $p = 0.19$).

When calculating joint specific power, the knee joint was found to be the main contributor to external work rate at all cadences with a joint specific power ranging from 34.6 to 77.1 W (corresponding to a relative contribution of 47 to 72 %) for 60 to 110 rpm, respectively (Fig. 2). Changing cadence had a significant effect on joint specific power (Fig. 2) with an increase in cadence leading to an increase in relative knee joint contribution [$p < 0.05$, $F(2,30) = 126.82$] and a decrease in relative hip joint contribution [$p < 0.05$, $F(3,36) = 174.06$]. When analysing knee flexion and extension phases of pedalling, the main contributor to external work rate was knee extension for all cadences (Fig. 3). We found a significant increase for knee joint contribution in both the flexion and extension phases [$p < 0.05$, $F(2,29) = 99.81$ and $F(2,24) = 77.36$, respectively]. Post-hoc analysis showed a continuous increase in knee joint extension from low to high cadences, while knee joint flexion showed a delayed increase (80–110 rpm) (Fig. 3).

Muscle oxygenation results are summarized in Table 2. There was no main effect of muscle on HHb, indicating that the changes in HHb during exercise were similar for VL and VM [$p = 0.95$, $F(1,16) = 2.01$]. There was a main effect of cadence on HHb [$p < 0.05$, $F(1,23) = 28.71$] showing that an increase in cadence led to increased HHb. Since no interaction effect was found [$p = 0.19$, $F(2,31) = 1.74$], the effect of cadence on HHb was similar for both muscles. Post-hoc analysis showed a continuous increase in HHb from low to high cadences in both muscles (Table 2).

For StO_2 during exercise however, there was a main effect of muscle with a more pronounced desaturation in the VL as compared the VM ($p < 0.05$, $F(1,13) = 6.66$]. On average StO_2 values for VL were 5.85 ± 2.3 % lower than those for VM. There was a main effect of cadence on StO_2 as well, leading to a more pronounced desaturation with increasing cadence [$p < 0.05$, $F(2,28) = 24.96$]. No interaction effect was found [$p = 0.42$ $F(2,30) = 0.94$], indicating that the effect of cadence on desaturation was similar for both the VL and the VM muscles. Post-hoc analysis showed a continuous decrease in StO_2 from an increase in cadence from 70 rpm and above in both muscles (Table 2).

A main effect of muscle was found for changes in tHb during exercise, with on average, tHb values in the VL that were 7.38 ± 1.7 μMol higher than those in the VM [$p < 0.05$, $F(1,16) = 6.76$]. However, there was no main effect of cadence [$p = 0.24$, $F(1,21) = 1.52$] and no interaction effect [$p = 0.17$, $F(2,24) = 1.94$], indicating that the change in cadence did not affect blood volume changes in either of the muscles.

A main effect of muscle was found for changes in O_2Hb during exercise, with on average, O_2Hb values in the VL that were 7.50 ± 1.9 μMol higher than those in the VM [$p < 0.05$, $F(1,16) = 14.61$]. There was a main effect of cadence leading to a decrease in O_2Hb with increasing cadence [$p < 0.05$, $F(1,23) = 17.77$]. There was no interaction effect [$p = 0.17$, $F(2,29) = 1.92$], thus the effect of cadence on O_2Hb during exercise was similar for both muscles.

With regard to mVO_2 (in % ischemic calibration/second) during exercise there was a main effect of muscle with the VL showing a higher mVO_2 compared to the VM [$p < 0.05$, $F(1,8) = 37.73$]. On average, mVO_2 in the VL

Table 2 Group mean and standard error for muscle oxygen consumption (mVO_2), tissue saturation index (StO_2), deoxyhaemoglobin (HHb), total haemoglobin (tHb) and oxyhaemoglobin (O_2Hb)

	Rest	60 rpm	70 rpm	80 rpm	90 rpm	100 rpm	110 rpm
mVO_2 $^{1}_{VL(ml/100\ g/min)}$	0.13 ± 0.01	3.77 ± 0.51[a]	4.00 ± 0.50	3.93 ± 0.52	4.05 ± 0.55	4.11 ± 0.51	4.73 ± 0.53
mVO_2 $_{VM(ml/100\ g/min)}$	0.12 ± 0.01	2.04 ± 0.24[a]	2.11 ± 0.25	2.10 ± 0.23	2.10 ± 0.24	2.12 ± 0.25	2.28 ± 0.25
StO_2 $^{2}_{VL(\%)}$	61.3 ± 3.9	55.18 ± 2.01[a]	54.12 ± 2.22	52.70 ± 2.21*	51.27 ± 2.53*	50.11 ± 2.48*	47.96 ± 2.80*[,b,c]
StO_2 $_{VM(\%)}$	66.4 ± 0.6	60.80 ± 1.19[a]	59.50 ± 1.31	58.04 ± 1.46*	56.94 ± 1.55*	55.72 ± 1.63*	54.75 ± 1.65*[,b,c]
HHb $_{VL(\Delta\mu Mol)}$	n/a	5.80 ± 1.42[a]	6.91 ± 1.42*	7.97 ± 1.50*	8.97 ± 1.67*	9.74 ± 1.75*	10.31 ± 1.81[b]
HHb $_{VM(\Delta\mu Mol)}$	n/a	5.38 ± 1.65[a]	6.75 ± 1.96*	8.14 ± 2.18*	9.04 ± 2.25*	10.12 ± 2.32*	10.93 ± 2.38[b]
tHb $_{VL(\Delta\mu Mol)}$	n/a	15.05 ± 1.48[a]	16.13 ± 1.55	16.33 ± 1.56	16.26 ± 1.61	16.21 ± 1.63	15.58 ± 1.77
tHb $_{VM(\Delta\mu Mol)}$	n/a	8.40 ± 1.71[a]	8.85 ± 1.67	8.95 ± 1.69	8.65 ± 1.84	8.41 ± 1.92	7.99 ± 1.94
O_2Hb $_{VL(\Delta\mu Mol)}$	n/a	9.25 ± 1.36[a]	9.23 ± 1.48	8.36 ± 1.53	7.30 ± 1.72*	6.47 ± 1.81*	5.27 ± 2.01[b,c]
O_2Hb $_{VM(\Delta\mu Mol)}$	n/a	3.02 ± 1.75	2.11 ± 2.07	0.81 ± 2.27	−0.39 ± 2.39*	−1.71 ± 2.46*	−2.94 ± 2.45[b,c]

* Indicates a significant change from previous cadence ($p < 0.05$)

[a] Cadence controlled test is different from rest ($p < 0.05$)

[b] Main effect of cadence ($p < 0.05$)

[c] Difference between muscles ($p < 0.05$)

[1] Due to the strict quality criteria for mVO_2 calculations a total of nine complete datasets were included in the two-way ANOVA for mVO_2

[2] Due to a malfunction with the NIRS equipment, only 14 participants were included for calculation of StO_2 for the VL

was 5.3 ± 0.9 % ischemic calibration/second for the VL and VM respectively. There was a main effect of cadence [$p < 0.05$, $F(5,40) = 2.72$], leading to increased mVO_2 with increased cadence. There was also an interaction effect [$p < 0.05$, $F(5,40) = 5.51$], thus the effect of cadence was different for the two muscles. As can be seen in Fig. 4, post hoc analysis showed that the VL mVO_2 increased significantly at 110 compared to 60 rpm [$p < 0.05$, $F(1,9) = 7.87$], whereas no change in mVO_2 was found for VM [$p = 0.28$, $F(2,18) = 1.38$].

When the effect of cadence on VL mVO_2 as a function of knee joint specific power was investigated, a quadratic model was superior to a linear model, thus indicating a non-linear relationship (LogLikelihood ratio = 11.7; $p < 0.05$) (Fig. 5a). When we combined mVO_2 of the VL and VM, we found only a tendency for a main effect of cadence [$p = 0.07$ $F(3,20) = 2.73$]. When the combined mVO_2 of the VL and VM, was expressed as a function of knee joint power, there was no difference between the linear and quadratic models, thus indicating a linear relationship (LogLikelihood ratio = 1.44; $p = 0.23$) (Fig. 5b).

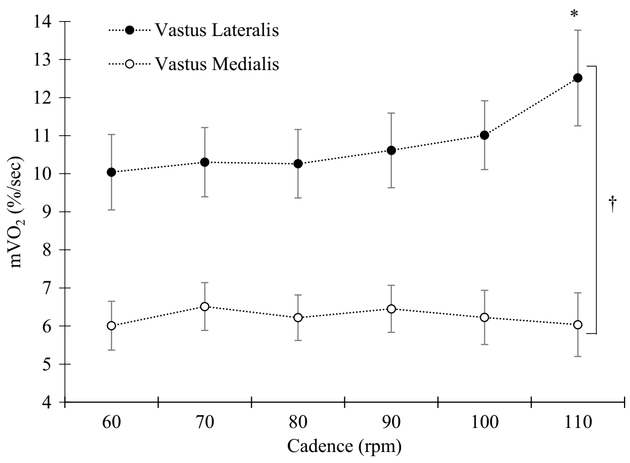

Fig. 4 Group mean and standard error for muscle VO_2 for the VL (*filled circles*) and VM (*open circles*) presented as change in percent of ischemic calibration per second. *Asterisk* indicate a significant increase in mVO_2 when compared to the 60 rpm condition. *Dagger* indicate a significant difference in the effect of cadence between the VL and VM muscles

Discussion

The purpose of this study was to investigate the effect of cadence on joint specific power contribution and local muscle deoxygenation and mVO_2 in the VL and VM during constant load cycling exercise. The main finding was that increased cadence led to an increase in mVO_2 the VL but not the VM. However, when combined, the VL and VM mVO_2 showed a linear relationship with knee joint specific power. Additionally, we found a significant effect of increasing cadence, leading to increased knee joint specific power and deoxygenation and a decreased StO_2 and O_2Hb in the VL and VM. This is the first study to report the effect of cadence on StO_2 in more than one muscle during constant load cycling exercise.

As expected there was an effect of increasing cadence on pVO_2 which was increased at 80 rpm compared to 60 rpm. Also further increases in cadence above 80 rpm led to an increase in pVO_2. Blood lactate and heart rate was also increased with increasing cadence. All of which are in accordance with previous reports (Foss and Hallen 2004).

We found knee extension was the main power producing joint action and we found an increasing knee joint and decreasing hip joint contribution with increasing cadence. As expected, we found that the ankle contribution to external power was not affected by cadence (Bini et al. 2010a; Elmer et al. 2011; Ericson 1988; Mornieux et al. 2007).

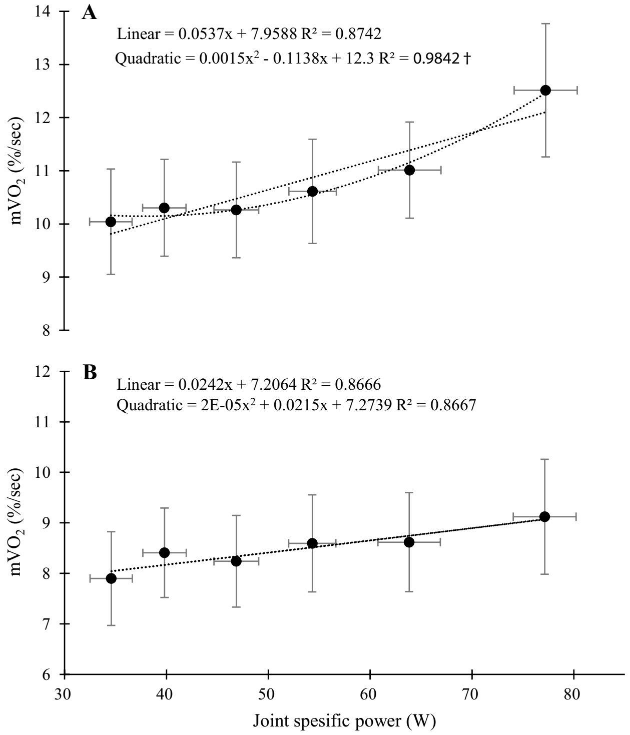

Fig. 5 **a** Mean and standard error for mVO_2 of the VL presented as a function of knee joint specific power (W). Linear and quadratic models are fitted to the data. *Dagger* indicate a significant better fit of the quadratic equation. **b** Mean and standard error for mVO_2 of the VL and VM combined presented as a function of knee joint specific power (W). Linear and quadratic models are fitted to the data

The effect of cadence was in accordance with our hypothesis and reports by Elmer et al. (2011) and Mornieux et al. (2007). Ericson (1988) reported no effect of cadence, but this might be due to the use of both increasing work rate and cadence as increase in work rate has been shown to increase relative hip contribution at low to moderate work rates (Skovereng et al. 2015). Elmer et al. (2011) reported hip extension to be the main contributor to external power production, but this may be due to a much larger work rate used. Additionally, knee extension has been reported to be the main contributor at low to moderate work rates comparable to the ones used in the present study (Skovereng et al. 2015). We found that increasing cadence from 60 to 110 rpm led to a shift in joint specific power with a total increase in knee joint power of ~43 W (i.e. ~25 % of external load) and a corresponding decrease in hip joint power. When calculating joint power from inverse dynamics, it is not possible to quantify the amount of power transfer from bi-articular muscles. Additionally, the power transfer to the hip from the upper body has been estimated to ~6 % (Broker and Gregor 1994) and a change in hip transfer power may account for part of the shift in joint specific power from the hip to the knee as it was not quantified in the present study.

HHb increased in both the VL and VM muscles for all cadences increases except from 100 to 110 rpm. This is consistent with the findings of Boone et al. (2014) who reported increased HHb at 100 rpm compared to 50 rpm during an incremental protocol. Takaishi et al. (2002) reported increased bottom peak HHb during the pedal stroke at 85 rpm compared to 50 rpm. However, Zorgati et al. (2013) and Kounalakis and Geladas (2012) reported no difference in deoxygenation between 40–100 and 40–80 rpm, respectively. However, 40 rpm is substantially lower than the 60 rpm that was used in the present study and also lower that the 50 rpm that was used by Takaishi et al. (2002) and it is possible that deoxygenation is higher at very low cadences. Also, work rate at baseline reported by Boone et al. (2014) is very low so it is difficult to compare with the results of the present study.

Total haemoglobin was increased at 60 rpm compared to rest, but there was no effect of cadence for the VL or the VM. This is coherent with the findings of Takaishi et al. (2002) who reported no effect of cadence on blood volume. Contrary, Kounalakis and Geladas (2012) found decreased tHb at 80 compared to 40 rpm, but this was during a much longer duration than in the present study.

StO_2 decreased following increased cadence for both the VL and VM (Table 2). To the best of our knowledge, only one study has reported the effect of cadence on StO_2 during constant external work rate cycling exercise. Kounalakis and Geladas (2012) reported no difference in StO_2 at 40 and 80 rpm. Kounalakis et al. (2008) showed increased StO_2 at 80 compared to 40 rpm, but used different external work rates to yield a similar p$\dot{V}O_2$. We found decreased O_2Hb following increased cadence, which is in accordance with previous reports (Kounalakis and Geladas 2012).

Since increasing cadence at a constant external work rate increases p$\dot{V}O_2$, our findings of increased HHb and decreased O_2Hb and StO_2 is to be expected considering previous reports on increasing external work rate (Peltonen et al. 2013). There is a lack of studies investigating the effect of cadence on StO_2, HHb, O_2Hb and tHb during constant external work rate cycling exercise and this is to the best of our knowledge the first study to report StO_2 and O_2Hb in multiple muscles during constant load exercise.

Considering m$\dot{V}O_2$, there is a difference in the effect of cadence in the m$\dot{V}O_2$ of the VL and VM at a cadence of 110 rpm compared to 60 rpm and no main effect of cadence, a finding that was unexpected. Given the increase in knee extension power there should also be an increase in m$\dot{V}O_2$ of the vasti. The absolute resting m$\dot{V}O_2$ was ~0.13 and ~0.12 ml/100 g/min for the VL and VM respectively in the present study (Table 2) which is comparable to previous reports using NIRS derived m$\dot{V}O_2$ in the forearm (Van Beekvelt et al. 2001b). Peak absolute m$\dot{V}O_2$ was ~4.2 and ~2.3 ml/100 g/min for the VL and VM respectively in the present study (Table 2). This is comparable to that measured in the forearm (Van Beekvelt et al. 2001b).

The finding that increasing cadence did not affect VM m$\dot{V}O_2$ can be due to changes resulting from muscle heterogeneity (Zorgati et al. 2013). Heterogeneity within muscles has been shown for m$\dot{V}O_2$ following increasing work rate (Vogiatzis et al. 2015) and also larger heterogeneity for deoxygenation at higher cadences (Zorgati et al. 2013). Since we only used one optode on the VL and VM respectively in the present study, there is a possibility that such heterogeneity has influenced our results. To the best of our knowledge, there have been no reports on the effect of cadence on intra or inter-muscular heterogeneity of m$\dot{V}O_2$, but based on the reports on deoxygenation it may exists and this should be a focus for future studies.

A previously reported difference between the VL and VM is a larger proportion of fast twitch fibres in the VL compared to the VM (Johnson et al. 1973). With the increase in pedal rate, the muscle contraction velocity must increase and a muscle with a larger proportion of fast twitch fibres would have a greater potential for force generation at high contraction speeds. The larger proportion of fast-twitch fibres reported for the VL can contribute to the finding of increasing m$\dot{V}O_2$ in the VL and not the VM at a high cadence.

From 60 to 100 rpm there seem to be no increase in the m$\dot{V}O_2$ of the leg muscles but we still see an increase

in pVO_2. Part of the increase in pVO_2 has been reported to originate in areas other than the legs (e.g., stabilization of the upper body). Umberger et al. (2006) showed increased pVO_2 as cadence increased, but the energy expenditure of the leg muscles did not increase until cadence exceeded 100 rpm. This is in accordance with the results of the present study.

The response of HHb, O_2Hb and StO_2 are to be expected when considering joint specific power. The increase in knee extension power indicates that the VL and VM will likely work harder. Correspondingly, the increased HHb and decreased O_2Hb and StO_2 have previously been shown following increasing work rate (Boone et al. 2014), which has also been shown to increase knee extension power and VL mVO_2 (Skovereng et al. 2015). The findings by Kounalakis and Geladas (2012), who reported no difference in StO_2 at 40 and 80 rpm, contrast what you would expect from the results on knee joint power. However, Kounalakis and Geladas (2012) used a long duration protocol and the participants were not cyclists. Since fitness status has been shown to influence the effect of cadence on EMG, it can also be an influencing factor for oxygenation and due to the scarce amount of literature, should be investigated in future studies.

There is an increase in both joint specific power and mVO_2 following and increase in cadence. An increase in increase in knee extension power would therefore to expected seeing as the VL are work harder, and therefore have a higher mVO_2. However, whereas knee joint power increased with most cadence increases, the increase in mVO_2 was only significant at 110 rpm compared to 60 rpm. Based on in vitro studies, a linear relationship between work rate and oxygen consumption can be expected (Fenn 1924). However, it is very difficult to measure the work rate of individual muscles in vivo. Joint specific power does not reflect the work rate of individual muscles, but of muscle groups and this leaves potential for differences between muscles that work on the same joint. Differences between muscles are evident from the different effect of cadence on VL and VM mVO_2. Even though the VL and VM are affected differently by cadence, the combined mVO_2 of the VL and VM displays a linear relationship with knee joint specific power. Although we cannot conclude that the slope of the combined VL and VM mVO_2 to knee joint work rate relationship in the present study is representative for the actual muscle power-VO_2 relationship, it still indicates that the two individual muscles together generate the response seen at the joint.

The AO method was used to calculate mVO_2 in the present study and this method relies on a constant blood volume within the measured tissue during the periods used for mVO_2 calculation. This prerequisite is not always met and therefore a procedure for correction has been developed (Ryan et al. 2012). After manually checking the NIRS signal from the AOs, blood volume was not always constant and thus the NIRS signal was corrected for changes in blood volume prior to mVO_2 calculation (Ryan et al. 2012).

Since we were interested in mVO_2, we tried to minimize the anaerobic part of the metabolism. We used a low to moderate intensity in the present study, and additionally, measurements with corresponding blood lactate above 4 mMol/l and RER values above one were excluded. Blood lactate does have a lag time before it is detectable in the blood of the fingertip, so local anaerobic processes could have occurred. It is possible that there are differences in the anaerobic metabolism in the VL and VM, despite our attempts at minimizing the anaerobic contribution. This could be one reason that we see differences in mVO_2 of the VL and VM.

Taken together, we have taken precautions in order to obtain the reliable mVO_2 values from NIRS and the AO method. However, the finding of a relatively large difference in VL and VM mVO_2 was unexpected. This was also the case with the discrepancy between HHb and mVO_2. Given the large increase in knee extension power, a bigger increase in vasti mVO_2 would be expected. We achieved quite good signal quality (Fig. 1) on most measurements. However, due to our criteria of a minimum R^2 of 0.98, we had to exclude some of the participants during the analysis of the mVO_2 data because of data quality for the 110 rpm condition. We still had sufficient participants (10 and 11 for the VL and VM respectively and 9 with a complete data set for both muscles) and additional analysis (unreported) excluding the 110 rpm condition showed findings coherent with those reported in this paper. However, due to the minimal literature using the method on the lower limbs, we recommend future investigators try to recreate our study in order to verify our findings on mVO_2.

We found no differences in skinfold thickness between VL and VM and, due to the different NIRS systems, mVO_2 was expressed as change in percent of physiological range obtained from individual ischemic calibration (Brizendine et al. 2013), so any confounding of NIRS measurements, and thus mVO_2 (van Beekvelt et al. 2001a), should be minimized.

In conclusion the present study demonstrates a significant effect of increasing cadence leading to increased knee joint specific power and a corresponding increases in HHb and decrease in StO_2 and O_2Hb. Increasing cadence also lead to increased VL mVO_2 but no effect of cadence was seen for the VM. The differences in the effect of cadence on mVO_2 in the VL and VM shows that

differences between two mono-articular knee extensors occur when cyclists change cadence at a constant external work rate in cycling.

Open Access This article is distributed under the terms of the Creative Commons Attribution 4.0 International License (http://creativecommons.org/licenses/by/4.0/), which permits unrestricted use, distribution, and reproduction in any medium, provided you give appropriate credit to the original author(s) and the source, provide a link to the Creative Commons license, and indicate if changes were made.

References

Ansley L, Cangley P (2009) Determinants of optimal cadence during cycling. Eur J Sport Sci 9:61–85. doi:10.1080/17461390802684325

Bini RR, Diefenthaeler F (2009) Mechanical work and coordinative pattern of cycling: a literature review. Kinesiology 41:25–39

Bini RR, Rossato M, Diefenthaeler F, Carpes FP, dos Reis DC, Moro ARP (2010a) Pedaling cadence effects on joint mechanical work during cycling. Isokinet Exerc Sci 18:7–13. doi:10.3233/Ies-2010-0361

Bini RR, Tamborindeguy AC, Mota CB (2010b) Effects of saddle height, pedaling cadence, and workload on joint kinetics and kinematics during cycling. J Sport Rehabil 19:301–314

Boone J, Barstow TJ, Celie B, Prieur F, Bourgois J (2014) The impact of pedal rate on muscle oxygenation, muscle activation and whole-body VO during ramp exercise in healthy subjects. Eur J Appl Physiol. doi:10.1007/s00421-014-2991-x

Brizendine JT, Ryan TE, Larson RD, McCully KK (2013) Skeletal muscle metabolism in endurance athletes with near-infrared spectroscopy. Med Sci Sports Exerc 45:869–875. doi:10.1249/MSS.0b013e31827e0eb6

Broker JP, Gregor RJ (1994) Mechanical energy management in cycling-source relations and energy-expenditure. Med Sci Sports Exerc 26:64–74

DeLorey DS, Kowalchuk JM, Paterson DH (2003) Relationship between pulmonary O_2 uptake kinetics and muscle deoxygenation during moderate-intensity exercise. J Appl Physiol 95:113–120. doi:10.1152/japplphysiol.00956.2002

Elmer SJ, Barratt PR, Korff T, Martin JC (2011) Joint-specific power production during submaximal and maximal cycling. Med Sci Sports Exerc 43:1940–1947. doi:10.1249/MSS.0b013e31821b00c5

Ericson M (1988) Mechanical muscular power output and work during ergometer cycling at different loads and speeds. Eur J Appl Physiol 57:382–387

Ettema G, Loras HW (2009) Efficiency in cycling: a review. Eur J Appl Physiol 106:1–14. doi:10.1007/s00421-009-1008-7

Ettema G, Loras H, Leirdal S (2009) The effects of cycling cadence on the phases of joint power, crank power, force and force effectiveness. J Electromyogr Kinesiol 19:e94–e101. doi:10.1016/j.jelekin.2007.11.009

Fenn WO (1924) The relation between the work performed and the energy liberated in muscular contraction. J Physiol 58:373–395

Ferreira LF, Lutjemeier BJ, Townsend DK, Barstow TJ (2006) Effects of pedal frequency on estimated muscle microvascular O_2 extraction. Eur J Appl Physiol 96:558–563. doi:10.1007/s00421-005-0107-3

Foss O, Hallen J (2004) The most economical cadence increases with increasing workload. Eur J Appl Physiol 92:443–451. doi:10.1007/s00421-004-1175-5

Hug F, Dorel S (2009) Electromyographic analysis of pedaling: a review. J Electromyogr Kinesiol 19:182–198. doi:10.1016/j.jelekin.2007.10.010

Hull ML, Jorge M (1985) A method for biomechanical analysis of bicycle pedalling. J Biomech 18:631–644

Johnson MA, Polgar J, Weightman D, Appleton D (1973) Data on the distribution of fibre types in thirty-six human muscles. An autopsy study. J Neurol Sci 18:111–129

Kounalakis SN, Geladas ND (2012) Cardiovascular drift and cerebral and muscle tissue oxygenation during prolonged cycling at different pedalling cadences. Appl Physiol Nutr Metab 37:407–417. doi:10.1139/h2012-011

Kounalakis SN, Keramidas ME, Nassis GP, Geladas ND (2008) The role of muscle pump in the development of cardiovascular drift. Eur J Appl Physiol 103:99–107. doi:10.1007/s00421-007-0662-x

Leirdal S, Ettema G (2011) The relationship between cadence, pedalling technique and gross efficiency in cycling. Eur J Appl Physiol 111:2885–2893. doi:10.1007/s00421-011-1914-3

Lucia A, San Juan AF, Montilla M, CaNete S, Santalla A, Earnest C, Perez M (2004) In professional road cyclists, low pedaling cadences are less efficient. Med Sci Sports Exerc 36:1048–1054

Martin JC, Brown NA (2009) Joint-specific power production and fatigue during maximal cycling. J Biomech 42:474–479. doi:10.1016/j.jbiomech.2008.11.015

McGhie D, Ettema G (2011) The effect of cadence on timing of muscle activation and mechanical output in cycling: on the activation dynamics hypothesis. J Electromyogr Kinesiol 21:18–24. doi:10.1016/j.jelekin.2010.04.007

Mornieux G, Guenette JA, Sheel AW, Sanderson DJ (2007) Influence of cadence, power output and hypoxia on the joint moment distribution during cycling. Eur J Appl Physiol 102:11–18. doi:10.1007/s00421-007-0555-z

Murias JM, Spencer MD, Keir DA, Paterson DH (2013) Systemic and vastus lateralis muscle blood flow and O_2 extraction during ramp incremental cycle exercise. Am J Physiol Regul Integr Comp Physiol 304:R720–R725. doi:10.1152/ajpregu.00016.2013

Neptune RR, Kautz SA, Hull ML (1997) The effect of pedaling rate on coordination in cycling. J Biomech 30:1051–1058

Peltonen JE et al (2013) Alveolar gas exchange, oxygen delivery and tissue deoxygenation in men and women during incremental exercise. Respir Physiol Neurobiol 188:102–112. doi:10.1016/j.resp.2013.05.014

Ryan TE, Erickson ML, Brizendine JT, Young HJ, McCully KK (2012) Noninvasive evaluation of skeletal muscle mitochondrial capacity with near-infrared spectroscopy: correcting for blood volume changes. J Appl Physiol 113:175–183. doi:10.1152/japplphysiol.00319.2012

Sargeant AJ (2007) Structural and functional determinants of human muscle power. Exp Physiol 92:323–331. doi:10.1113/expphysiol.2006.034322

Sarre G, Lepers R, Maffiuletti N, Millet G, Martin A (2003) Influence of cycling cadence on neuromuscular activity of the knee extensors in humans. Eur J Appl Physiol 88:476–479. doi:10.1007/s00421-002-0738-6

Skovereng K, Ettema G, van Beekvelt M (2015) Local muscle oxygen consumption related to external and joint specific power. Hum Mov Sci 45:161–171. doi:10.1016/j.humov.2015.11.009

Takaishi T, Ishida K, Katayama K, Yamazaki K, Yamamoto T, Moritani T (2002) Effect of cycling experience and pedal cadence on the near-infrared spectroscopy parameters. Med Sci Sports Exerc 34:2062–2071. doi:10.1249/01.MSS.0000039304.91231.76

Umberger BR, Gerritsen KG, Martin PE (2006) Muscle fiber type effects on energetically optimal cadences in cycling. J Biomech 39:1472–1479. doi:10.1016/j.jbiomech.2005.03.025

van Beekvelt MC, Borghuis MS, van Engelen BG, Wevers RA, Colier WN (2001a) Adipose tissue thickness affects in vivo quantitative

near-IR spectroscopy in human skeletal muscle. Clin Sci (Lond) 101:21–28

Van Beekvelt MC, Colier WN, Wevers RA, Van Engelen BG (2001b) Performance of near-infrared spectroscopy in measuring local O(2) consumption and blood flow in skeletal muscle. J Appl Physiol 90:511–519

van Soest AJ, Schwab AL, Bobbert MF, van Ingen Schenau GJ (1993) The influence of the biarticularity of the gastrocnemius muscle on vertical-jumping achievement. J Biomech 26:1–8

Vogiatzis I, Habazettl H, Louvaris Z, Andrianopoulos V, Wagner H, Zakynthinos S, Wagner PD (2015) A method for assessing heterogeneity of blood flow and metabolism in exercising normal human muscle by near-infrared spectroscopy. J Appl Physiol 118:783–793. doi:10.1152/japplphysiol.00458.2014

Zajac FE (2002) Understanding muscle coordination of the human leg with dynamical simulations. J Biomech 35:1011–1018. doi:10.1016/S0021-9290(02)00046-5

Zorgati H, Collomp K, Amiot V, Prieur F (2013) Effect of pedal cadence on the heterogeneity of muscle deoxygenation during moderate exercise. Appl Physiol Nutr Metab 38:1206–1210. doi:10.1139/apnm-2012-0504
